# Chromosome-level genome assembly of *Acrossocheilus fasciatus* using PacBio sequencing and Hi-C technology

**DOI:** 10.1038/s41597-024-02999-6

**Published:** 2024-02-03

**Authors:** Jianbo Zheng, Jianhu Jiang, Qianlong Rui, Fei Li, Shili Liu, Shun Cheng, Meili Chi, Wenping Jiang

**Affiliations:** 1 Key Laboratory of Genetics and Breeding, Zhejiang Institute of Freshwater Fisheries, Huzhou, China; 2 College of Biological and Environmental Sciences, Zhejiang Wanli University, Ningbo, China

**Keywords:** Animal breeding, Chromosomes

## Abstract

*Acrossocheilus fasciatus* (Cypriniformes, Cyprinidae) is emerged as a newly commercial stream fish in the south of China with high economic and ornamental value. In this study, a chromosome-level reference genome of *A. fasciatus* was assembled using PacBio, Illumina and Hi-C sequencing technologies. As a result, a high-quality genome was generated with a size of 879.52 Mb (accession number: JAVLVS000000000), scaffold N50 of 32.7 Mb, and contig N50 of 32.7 Mb. The largest and smallest scafford was 60.57 Mb and 16 kb, respectively. BUSCO analysis showed a completeness score of 98.3%. Meanwhile, the assembled sequences were anchored to 25 pseudo-chromosomes with an integration efficiency of 96.95%. Additionally, we found approximately 390.91 Mb of repetitive sequences that accounting for 44.45% of the assembled genome, and predicted 24,900 protein-coding genes. The available genome reported in the present study provided a crucial resource to further investigate the regulation mechanism of genetic diversity, sexual dimorphism and evolutionary histories.

## Background & Summary

**Fig. 1:**
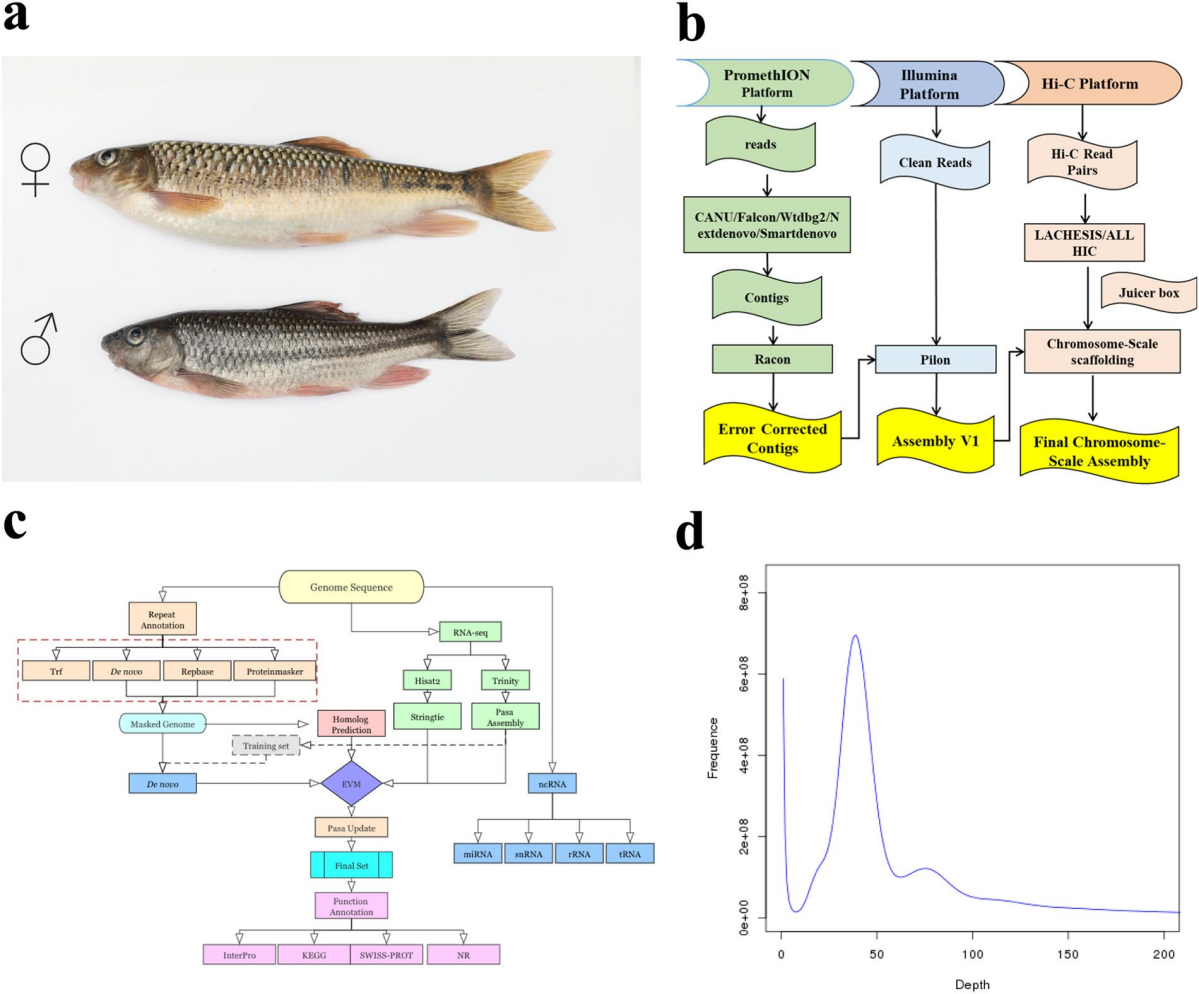
Workflow of the genome assembly and survey analysis in *A. fasciatus*. (**a**) A picture of female and male *A*. *fasciatus*. ♂ indicates male individual, and ♀ indicates female individual. (**b**) The work flow used for genome sequencing. (**c**) Flow chart of the genome annotation. (**d**) The 17-mer distribution for the genome size estimation.

The genus *Acrossocheilus* belongs to Barbinae, Cyprinidae, and is composed of approximately 26 species, which are mainly native in Laos, Vietnam, and China^1^. Meanwhile, these groups exhibit diversiform morphological characteristics and ecological habits, providing a great model for investigating species origin and geographical distribution of freshwater fish^2^. In addition, it’s flesh is tender, delicious and contains highly polyunsaturated fatty acids (PUFA), possessing a considerable market value. Recently, the freshwater grouper *A. fasciatus* has become a commercially emerging aquaculture fish due to its nutritive and ornamental value^3^. Moreover, as an omnivorous fish, the growth of *A. fasciatus* requires to feed with moss and other algae plants, which can inhibit the rankness of these aquatic plants, thus playing a role in ecological balance. Previous studies of *A. fasciatus* have primarily focused on its embryos and larval development, gonad histological characteristics, phylogenetic relationships, population structure,and artificial breeding^4–6^. On the other hand, *A. fasciatus* represents significant difference in growth rate and body size between males and females, with females growing faster than males (Fig. 1a), indicating all-female breeding is of high commercial value in aquaculture^7^. However, our knowledge of *A. fasciatus* on genetic and evolutionary mechanisms have been limited due to lack of genetic resources and genomic information. In this study, we employed an integrated strategy of PacBio, Illumina and Hi-C sequencing technologies to assemble a high-quality genome in a size of 879.52 Mb with scaffold N50 of 32.7 Mb (Fig. 1b,c). We believe that this high-quality of chromosomal-level genome data will provide valuable resources for breeding programs and evolutionary investigation.

## Methods

### Sample collection and nucleic acid extraction

Mature and healthy *A. fasciatus* were obtained from Zhejiang institute of freshwater fisheries in Huzhou, Zhejiang province, China. Muscle tissues from adult female *A. fasciatus* was prepared for DNA extraction with SDS lysis method, while ovary, kidney, brain, testis, skin, and gill were collected for total RNA extraction using a TRIzoL kit following the manufacturer’s protocol. Herein, the high-quality gDNA was used for genome sequencing, and total RNA isolated from all tissues were used for transcriptome sequencing.

### Library construction and genome sequencing

For the Illumina platform (NEB, USA), a paired-end library with an insertion size of 350 bp was generated using NEB Next® Ultra™ DNA Library Prep Kit following manufacturer’s recommendations. As a result, a total of 41 Gb Illumina short-reads (coverage of 47.56X, Table 1) with paired-end 150 bp were generated. Simultaneously, HiFi SMRTbell Libraries was prepared using SMRTbell Express Template Prep Kit 2.0 for long-read sequencing with insert size of 20 kb on Pacbio platform. In briefly, gDNA was sheared to 6–20 kb fragments using the g-TUBE, and the ssDNA overhangs were removed with *Exo VII*. Then DNA damage was repaired for Blunt-End ligation, and large insert SMRTbell libraries were constructed after size selection to prepare for sequencing use DNA Sequencing Reagent Kit. For the PacBio platform, approximately 32 Gb PacBio reads (37.12X coverage, Table 1) were obtained with the longest read of 47.52 kb and the N50 length of 14.56 kb.

**Table 1:** Statistics of the sequencing data for the *A. fasciatus* genome assembly.

Library types	Insert size (bp)	Raw data (Gb)	Clean data (Gb)	Read length (bp)	Sequence coverage (X)
**Illumina reads**	350	41.00	36.72	150	47.56
**PacBio reads**	20,000	32.66	22.83	14,447	37.12
**Hi-C reads**	350	86.32	76.58	150	—
**RNA reads**	350	41.18	37.51	150	—
**Total**	—	201.16	173.64	—	—

### Genome size estimation and assembly

Herein, clean data generated from Illumina sequencing were subjected to k-mer analysis to estimate the genome size, heterozygosity, and the proportion of repetitive sequences in *A. fasciatus*. Based on 17-mer frequency distribution using Jellyfish v2.3.0^8^ and GenomeScope v2.0^9^, the genome size was estimated to be 862.9 Mb, with a heterozygosity ratio of 0.56% and repeat sequence ratio of 47.09% (Fig. 1d). The 32.66 Gb raw subreads from the PacBio Sequel platform were filtered out, and the remaining clean subreads were error-corrected by Canu (v1.5)^10^ and pre-assembled into contigs using FALCON software^11^. The assembled scaffolds were polished by Pilon (v1.22)^12^ with default parameters. The finally assembled genome was 879.52 Mb in size with 134 contigs and a contig N50 of 32.70 Mb (Table 2).

**Table 2:** Summary of the assembled genome for *A. fasciatus* genome.

	PacBio		Hi-C	
	Scaffold	Contig	Scaffold	Contig
Total number	134	134	194	115
Total length (bp)	879,520,627	879,520,627	879,520,627	879,528,527
Average_length (bp)	6,563,586	6,563,586	7,648,074	4,533,611
Max length (bp)	60,574,424	60,574,424	36,374,165	54,140,365
Min length (bp)	16,004	16,004	18,174	16,004
N50 length (bp)	32,702,747	32,702,747	22,576,242	33,132,389
N50 number	11	11	16	12
N90 length (bp)	11,417,557	11,417,557	5,356,806	26,858,976
N90 number	28	28	45	23

### Hi-C library preparation and sequencing

**Fig. 2:**
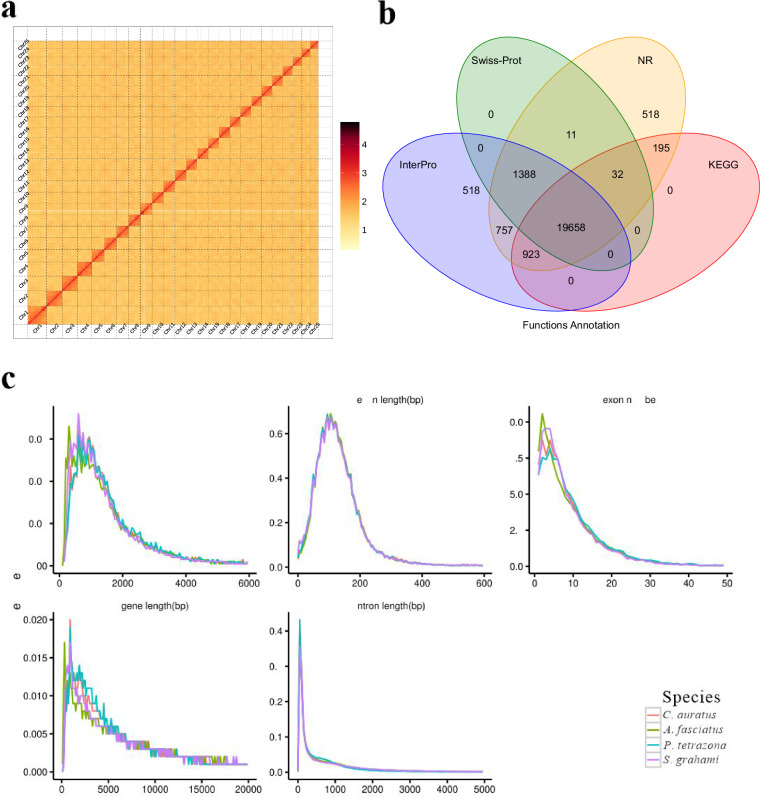
Chromosomal level assembly of *A. fasciatus* genome and functional annotation. (**a**) Heat maps of Hi-C assembly of *A. fasciatus*. The color bar indicates the logarithm of the strength of the contact density. (**b**) The Venn graph of the numbers of annotated genes with different databases. (**c**) The comparisons of different gene elements in *A. fasciatus* geneome with three other fish species.

The Hi-C libraries were constructed following the standard protocol described previously with certain modifications. Firstly, female muscle samples were cross-linked by 4% formaldehyde, and the fixed tissues were homogenised and centrifuged to collect the nuclei, then digested with *Mbo I* enzyme overnight at 37 °C. The proximal chromatin DNA was re-ligated using T4 ligase, and Biotin-labeled Hi-C samples were specifically enriched using magnetic beads. After adding A-tails to the fragment ends, Hi-C sequencing libraries were amplified by PCR and sequenced on Illumina HiSeq-2500 platform (PE 150 bp). For chromosome-level assembly, the raw Hi-C sequencing data were primarily filtered using Hi-C-Pro v2.8.0^13^, and the high-quality clean reads were aligned to the polished *A. fasciatus* genome using BWA (v0.7.10)^14^ with default parameters (samtools sort sample.sam–output-fmt BAM–o sample.sort.bam). Finally, 96.95% of the initial assembled sequences were anchored to 25 pseudo-chromosomes that ranged in size from 24.09 to 54.14 Mb (Fig. 2a, Table S1), and the total length of the genome assembly was 879.52 Mb with a contig N50 of 22.57 Mb, and scaffold N50 of 33.13 Mb (Table 2).

### Repetitive sequence annotation

Repeat elements in the *A. fasciatus* genome were annotated employing a combined methods of homology alignment and *de novo* searches. The homology-based blast was performed against the RepBase data base (http://www.girinst.org/repbase/)^15^ using Repeatmasker and repeatproteinmask software for known repeat elements. For *de novo* annotation, we firstly employed LTR_FINDER^16^, RepeatModeler^17^ and RepeatScout^18^ to bulid a *de novo* repeat library, and then was used to predict repeat elements using Repeatmasker with default parameters. Additionally, Tandem Repeats can be identified using Tandem Repeat Finder (TRF, http://tandem.bu.edu/trf/trf.html)^19^. In this study, we identified 390.91 Mb of repetitive sequences, accounting for 44.45% of the assembled genome (Table 3).

**Table 3:** Classification of the predicted repeat sequences in the genome of *A. fasciatus*.

	Denovo + Repbase		TE Proteins		Combined TEs	
	Length (bp)	% in Genome	Length (bp)	% in Genome	Length (bp)	% in Genome
DNA	62,039,748	7.05	3,890,780	0.44	63,377,571	7.21
LINE	12,947,711	1.47	17,737,914	2.02	24,472,470	2.78
SINE	402,411	0.05	0	0	402,411	0.05
LTR	295,839,499	33.64	14,401,516	1.64	296,730,746	33.74
Unknown	18,113,674	2.06	0	0	18,113,674	2.06
Total	380,053,264	43.21	36,025,623	4.10	382,870,399	43.53

Note: TE, transposable element; LINE, long interspersed nuclear elements; SINE, short interspersed nuclear elements; LTR, long terminal repeats.

### Gene prediction and functional annotation

Protein-coding genes were annotated through integrating three different strategies of homology, *de novo*, and transcriptome-based prediction methods. For homology-based gene prediction, the published protein sequences of *Sinocyclocheilus grahami*, *Puntius tetrazona* and *Carassius auratus* were aligned to the *A. fasciatus* genome assembly using BLAST^20^ and Genewise^21^ with default parameters. Five *de novo* programs, including Augustus^22^, GlimmerHMM^23^, SNAP^24^, GeneID^25^ and GENSCAN^26^, were used to predict coding regions in the repeat-masked assembly with default parameters. For the transcriptome-based annotation, the RNA-seq data were *de novo* assembled by Trinity (v2.1.1)^27^ and splicing variations were identified by PASApipeline (v2.4.1)^28^. Finally, a non-redundant reference gene set was established by merging the above three methods, resulting in a total of 24,900 protein-coding genes (Fig. 2b, Table 4). Simultaneously, we compared the gene parameters of different elements in *A. fasciatus* and three relative species (*S. grahami*, *C. auratus*, *P. tetrazona*), and the result showed a similar distribution of coding DNA sequence (CDS) length, exon length and number, intron length and mRNA length among the sequenced fish genomes (Fig. 2c).

**Table 4:** Statistical analysis of predicted protein-coding genes in *A. fasciatus* genome.

	Gene set	Number	Average transcript length (bp)	Average CDS length (bp)	Average exons per gene	Average exon length (bp)	Average intron length (bp)
**De novo**	Augustus	39,649	8,793.02	1,128.93	6.39	176.78	1,422.96
	GlimmerHMM	82,692	9,420.66	651.96	4.38	148.76	2,592.23
	SNAP	55,978	15,341.35	816.46	5.86	139.32	2,988.45
	Geneid	30,650	17,697.02	1,364.34	6.40	213.17	3,024.50
	Genscan	30,914	19,582.74	1,547.51	8.21	188.43	2,500.51
**Homolog**	Ptet	21,870	14,149.81	1,651.62	9.29	177.73	1,507.06
	Sgra	22,672	13,058.43	1,556.54	8.61	180.68	1,510.44
	Caur	23,079	13,629.20	1,623.40	9.01	180.28	1,499.77
**RNAseq**	PASA	27,840	13,376.06	1,383.89	8.23	168.18	1,658.94
	Transcripts	50,168	23,864.39	3,076.21	10.43	294.92	2,204.35
**EVM**		35,375	12,103.93	1,281.60	7.43	172.60	1,684.29
Pasa-update*		35,122	12,467.91	1,299.95	7.50	173.23	1,717.05
Final set*		24,900	15,927.24	1,627.71	9.56	170.21	1,669.96

Note: EVM, EVidenceModeler.

Furthermore, all predicted genes were functionally annotated using public biological function databases of SwissPro^29^, Nr (http://www.ncbi.nlm.nih.gov/protein), KEGG^30^ and InterPro^31^ and Pfam (http://pfam.xfam.org/). Overall, a total of 24,000 genes (96.40%) were successfully annotated with an average transcript length of 15,927.24 bp and an average CDS length of 1,627.71 bp (Table 5). In addition, non-coding RNAs (ncRNAs) were also annotated, and tRNAscan-SE (v2.0)^32^ was used to predict tRNAs, and Infernal (1.1)^33^ was used to identify rRNAs, snRNAs, and miRNAs. In total, 43,620 non-coding RNAs were predicted, including 17,604 tRNAs, 9,157 rRNAs, 2,606 miRNAs and 2,548 snRNAs (Table 6).

**Table 5:** Summary of functional annotation in *A. fasciatus* genome.

	Number	Percent(%)
**Total**	24,900	—
**Swissprot**	21,089	84.70
**Nr**	23,482	94.30
**KEGG**	20,808	83.60
**InterPro**	23,244	93.30
**GO**	16,364	65.70
**Pfam**	19,986	80.30
**Annotated**	24,000	96.40
**Unannotated**	900	3.60

**Table 6:** Statistics of annotated non-coding RNAs in the *A. fasciatus* genome assembly.

Type		Copy number	Average length (bp)	Total length (bp)	% of genome
**miRNA**		2,606	118.27	308,203	0.035042
**tRNA**		17,604	75.85	1,335,287	0.15
**rRNA**	**rRNA**	9,157	135.62	1,241,850	0.14
	**18S**	153	744.68	113,936	0.012954
	**28S**	442	422.19	186,608	0.021217
	**5.8S**	62	156	9,672	0.001100
	**5S**	8,500	109.60	931,634	0.11
**snRNA**	**snRNA**	2,548	146.53	373,349	0.042449
	**CD-box**	303	149.79	45,385	0.005160
	**HACA-box**	85	150.09	12,758	0.001451
	**splicing**	2,101	145.30	305,275	0.034709
	**scaRNA**	52	183.58	9,546	0.001085
	**Unknown**	7	55	385	0.000044

### Gene family construction

**Fig. 3:**
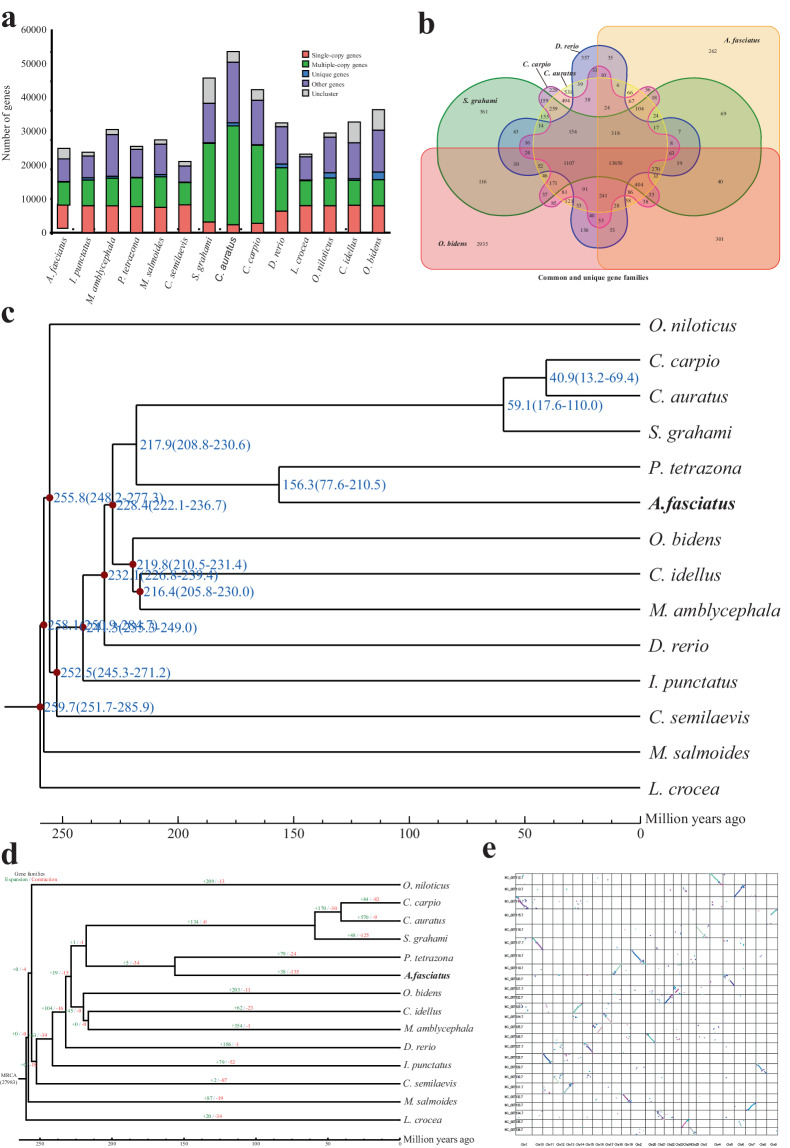
Comparative genomic analysis reveals phylogenetic positioning and genome evolution of *A. fasciatus*. (**a**) Statistics of orthologous gene families in 14 representative fish species. (**b**) Venn diagram of shared and unique orthologous gene families in *A. fasciatus* and four other teleosts. (**c**) Phylogenetic analysis and divergence time tree of *A. fasciatus* and other representative species. (**d**) Statistical analysis of contraction and expansion of gene families. (**e**) Comparative synteny analysis between *A. fasciatus* and zebrafish.

Firstly, the protein sequences of other 13 fish species, including *P. tetrazona*, *S. grahami*, *C. auratus*, *Opsariichthys bidenswere*, *Cyprinus carpio*, *Danio rerio*, *Ictalurus punctatus*, *Megalobrama amblycephala*, *Ctenopharyngodon idellus*, *Micropterus salmoides*, *Oreochromis niloticus*, *Cynoglossus semilaevis*, *Larimichthys crocea*, were downloaded from the public database. The low quality of sequences with less than 50 amino acids were then filtered out and only retained the longest predicted transcript per locus. Next, similarities between the protein sequences of all species were identified employing an all-to-all BLAST search with an e-value of 1e-5. Finally, orthologous gene clusters were performed using the the OrthoMCL^34^. In summary, we identified 27,983 gene families shared by *A. fasciatus* and the additional 13 species, and 10,524 gene families and 604 single-copy gene families were found in all species, respectively (Fig. 3a). Moreover, gene families from *A. fasciatus*, *O. bidens*, *S. grahami*, *D. rerio*, *C. carpio* and *C. auratus*, were further clustered, of which 13,850 gene families were shared by these fish species, and 262 gene families were specific to *A. fasciatus* (Fig. 3b). In addition, functional annotation was conducted for unique gene families in *A. fasciatus*, and revealed that Phosphatidylinositol signaling system, GABAergic synapse, Vitamin digestion and absorption, Lysine degradation, Synaptic vesicle cycle were enriched.

### Phylogenetic and evolutionary analysis

All single-copy gene families were subjected to multiple sequence alignment to generated a super alignment matrix by MUSCLE^35^, and a phylogenetic tree was constructed using RAxML^36^. Subsequently, the MCMCTree package in PAML^37^ was used to estimate divergence times. As expected, evolutionary analysis demonstrated that *A. fasciatus* and *P. tetrazona* were clustered into one clade, and their divergence time was estimated to be 156.3 million years ago (Fig. 3c). Furthermore, gene expansions and contractions were analyzed employing CAFE (v3.1)^38^ with default parameters based on the the divergence times and phylogenetic relationships. A total of 38 and 135 gene families significantly expanded and contracted in *A. fasciatus*, respectively (Fig. 3d). Finally, chromosome synteny between *A. fasciatus* and *D. rerio* were carried out using MCScanX software^39^, and visual diagram was generated by Circos. Synteny relationships analysis showed that the chromosomes of *A. fasciatus* displayed a high homology with the *D. rerio* chromosomes (Fig. 3e).

## Data Records

All sequencing data had been uploaded to NCBI database via the project PRJNA1012810. The genomic Illumina sequencing data were deposited in the Sequence Read Archive at SRR25949940^40^, SRR25949941^41^. The genomic PacBio sequencing data were deposited in the SRA at NCBI SRR25933437^42^. The transcriptomic sequencing data were deposited in the SRA at NCBI SRR25949840^43^, SRR25949841^44^, SRR25949842^45^, SRR25949843^46^, SRR25949844^47^, SRR25949845^48^. The Hi-C sequencing data were deposited in the SRA at NCBI SRR25947115^49^, SRR25947116^50^, SRR25947117^51^. The final chromosome assembly was deposited in the GenBank at NCBI with accession number: JAVLVS000000000^52^. The genome annotation file was also available in figshare^53^. The data for the gene family construction was available in the figshare database^54^.

## Technical Validation

### DNA quantification and qualification

DNA degradation and contamination was monitored on 1.5% agarose gels. DNA purity was checked using the NanoPhotometer® spectrophotometer (IMPLEN, CA, USA). DNA concentration was measured using Qubit® DNA Assay Kit in Qubit® 2.0 Fluorometer (Life Technologies, CA, USA).

### Quality control of raw sequencing data

To make sure reads reliable and without artificial bias (low quality paired reads, which mainly resulted from base-calling duplicates and adapter contamination) in the following analyses, raw data were firstly processed through a series of quality control (QC) procedures in-house C scripts. QC standards as the following: (1) Removing reads with ≥ 10% unidentified nucleotides (N); (2) Removing reads with >50% bases having phred quality <5.

### RNA quality evaluation

Before transcriptomes sequecing, the quality of total RNA from six tissues was validated. The concentration was measured by Qubit Fluorometr, and the integrity was detected using Aglient 2100 Bioanalyzer. Overall, RNAs samples with a total RNA amount ≧ 10 μg, RNA integrity ≧ 8, and rRNA ratio ≧ 1.5 were served as libraries construction.

### Evaluation of the assembled genome

The completeness and accuracy of the *A. fasciatus* genome assembly were evaluated by multiple methods. First, Benchmarking Universal Single-Copy Orthologs (BUSCO, v5.4.4)^55^ and Core Eukaryotic Genes Mapping Approach (CEGMA, v2.5)^56^ were used to assess the completeness of the assembled genome. The BUSCO results revealed that 98.3% of the complete BUSCOs and 0.7% of the fragmented BUSCOs were found in 3640 single-copy orthologs of actinopterygii_odb10, and 1.0% of BUSCOs was missing. Moreover, CEGMA evaluation showed that 96.77% (240/248) core eukaryotic genes (CEGs) were obtained. In addition, Merqury (v1.3)^57^ was ran to evaluate the accuracy of genome assembly, and a high quality value (QV) of 44.81 indicated that this assembly was of good quality. Taken together, these results suggested that the assembled *A. fasciatus* genome was of high quality at chromosome level.

### Supplementary information

Distributionof the assembled chromosomes of A. fasciatus.

## Data Availability

No special codes or scripts were used in this work, and Data processing was carried out based on the protocols and manuals of the corresponding bioinformatics software.
