# Supplementary material for: Chromosome-level genome assembly of Acrossocheilus fasciatus using PacBio sequencing and Hi-C technology
Source: Sci Data. 2024 Feb 3;11:166. doi: 10.1038/s41597-024-02999-6 (PMC10838343; doi:10.1038/s41597-024-02999-6)
Supplement: Supplementary file 1 — Distributionof the assembled chromosomes of A. fasciatus. [file 41597_2024_2999_MOESM1_ESM.docx]

**Table S1. Distributionof the assembled chromosomes of *A. fasciatus*.**

| **Sequeues ID** | **Cluster Number** | **Sequeues Length** | **Sequeues ID** | **Cluster Number** | **Sequeues Length** |
| --- | --- | --- | --- | --- | --- |
| Chr1 | 5 | 54,140,365 | Chr14 | 3 | 32,012,820 |
| Chr2 | 2 | 46,213,822 | Chr15 | 3 | 31,766,482 |
| Chr3 | 3 | 44,241,033 | Chr16 | 2 | 31,711,980 |
| Chr4 | 2 | 42,276,777 | Chr17 | 3 | 31,331,109 |
| Chr5 | 5 | 36,778,695 | Chr18 | 2 | 31,261,039 |
| Chr6 | 7 | 36,035,181 | Chr19 | 5 | 31,164,815 |
| Chr7 | 2 | 35,217,714 | Chr20 | 4 | 30,964,945 |
| Chr8 | 8 | 34,506,577 | Chr21 | 3 | 30,257,955 |
| Chr9 | 4 | 34,284,056 | Chr22 | 4 | 29,774,110 |
| Chr10 | 4 | 33,733,945 | Chr23 | 13 | 26,858,976 |
| Chr11 | 3 | 33,372,652 | Chr24 | 3 | 24,870,528 |
| Chr12 | 7 | 33,132,389 | Chr25 | 3 | 24,096,624 |
| Chr13 | 4 | 32,661,377 |  |  |  |
| **Class** | **Scaffold Number** | | **Total Length** | | |
| place | 25 | | 852,665,966 (96.95%) | | |
| unplace | 90 | | 26,862,561 (3.05%) | | |
| total | 115 | | 879,528,527 | | |
